# Beverages in Rheumatoid Arthritis: What to Prefer or to Avoid

**DOI:** 10.3390/nu12103155

**Published:** 2020-10-15

**Authors:** Mrinalini Dey, Maurizio Cutolo, Elena Nikiphorou

**Affiliations:** 1 Institute of Life Course and Medical Sciences, University of Liverpool, Brownlow Hill, Liverpool L69 3BX, UK; 2 Department of Rheumatology, Aintree Hospital, Liverpool University Hospitals NHS Foundation Trust, Lower Lane L9 7AL, UK; 3 Research Laboratories and Academic Division of Clinical Rheumatology, Postgraduate School of Rheumatology, Department of Internal Medicine, University of Genova, IRCCS San Martino Polyclinic, 16126 Genoa, Italy; mcutolo@unige.it; 4 Centre for Rheumatic Diseases, King’s College London, London SE5 9RJ, UK; elena.nikiphorou@kcl.ac.uk

**Keywords:** rheumatoid arthritis, beverage, nutrition, microbiome

## Abstract

Background: The role of nutrition in the pathogenesis of rheumatic diseases, including rheumatoid arthritis (RA), has gained increasing attention in recent years. A growing number of studies have focussed on the diverse nutritional contents of beverages, and their possible role in the development and progression of RA. Main body: We aimed to summarise the current knowledge on the role of a range of beverages in the context of RA. Beverages have a key role within the mosaic of autoimmunity in RA and potential to alter the microbiome, leading to downstream effects on inflammatory pathways. The molecular contents of beverages, including coffee, tea, and wine, have similarly been found to interfere with immune signalling pathways, some beneficial for disease progression and others less so. Finally, we consider beverages in the context of wider dietary patterns, and how this growing body of evidence may be harnessed by the multidisciplinary team in patient management. Conclusions: While there is increasing work focussing on the role of beverages in RA, integration of discussions around diet and lifestyle in our management of patients remains sparse. Nutrition in RA remains a controversial topic, but future studies, especially on the role of beverages, are likely to shed further light on this in coming years.

## 1. Introduction

Rheumatoid arthritis (RA) is a chronic, systemic, immune-inflammatory disease, with a complex aetiology, including genetic, environmental, and endogenous triggers [1,2]. Factors such as cigarette smoking, infectious agents, environmental pollution, and chronic stress have been cited as possible triggers for the intense inflammatory response and production of pro-inflammatory mediators seen in RA [3,4,5,6]. In recent years, there has been increasing evidence for the role of nutrition in RA disease onset and activity, although this has failed to filter down to clinical awareness and practice as an adjunct to pharmacological treatments [7,8]. This includes the important role played by beverages and their rich nutritional content [2].

Dietary habits have important effects on human health, for example in hypertension, diabetes, and heart disease [9]. Beverages in particular are a source of a vast array of nutrients, from vitamins and minerals to fats and proteins. In addition to their practical necessity, they have been central to our functioning as humans on a psychosocial level throughout history, from wine consumption in Ancient Rome, to the introduction of afternoon tea in the 1800s, to our modern ritualistic morning coffee or weekend pint of beer with friends. Therefore, from a socio-cultural point of view, beverages serve more than to simply rehydrate or provide nutritional gains. It is therefore unsurprising that research on the role of beverages in the pathogenesis of RA has increased, especially over the last decade, and it stands to reason that clinicians can and should do more to harness this increasing body of evidence in disease management.

Despite being an important and relevant topic, nutrition remains poorly taught through most medical curriculums. The role of the dietician in the multidisciplinary team is important and recognised at least by some, but not always possible. In addition, the controversy surrounding the role of nutrition in RA and a generally poor understanding of the literature mean patients can receive conflicting and confusing information. Meanwhile, we continue to move towards an era of personalised medicine, which includes the consideration of dietary and nutritional needs, and their impact on the individual patient.

This narrative review aims to provide an overview of the evidence on the possible role of beverages in the aetiology and progression of RA, and how this may also be of help in the care of our patients. The review was performed through targeted searches in MedLine for each of the beverages discussed below, in the context of RA. Key references were identified by all authors, and were also crosschecked in recent reviews of nutrition in RA.

### 1.1. The Mosaic of Autoimmunity in RA and the Role of Nutrition

The “mosaic of autoimmunity” was a term originally coined by Shoenfeld and Isenberg in 1989, and refers to the interplay between genetic, hormonal, immunological, and environmental factors in the pathogenesis of autoimmune diseases, including RA [10]. In recent decades, our understanding of genetic factors in the development of autoimmune conditions has progressed remarkably. Studies in monozygotic twins have demonstrated very high levels (four time or higher) of concordance in not just RA, but other autoimmune conditions such as type 1 diabetes mellitus, systemic lupus erythematosus, and multiple sclerosis [11]. The discovery of human leukocyte antigen (HLA) associations in multiple diseases (e.g., HLA-DR4 and DR1 in RA) has added to our understanding of the genetic basis of disease. However, the incomplete correlation in genotype and disease expression even in monozygotic twins highlights the fact that aetiology is due to more than just genetics [9].

Indeed, several recent studies report on a potential link between dietary factors and alterations in epigenetic pathways, providing compelling insight into the possible effects of environmental factors on fundamental biological processes and aetiology of autoimmune diseases [12]. For example, among beverages, both tea and coffee have been suggested to play an important role in modulating disease risk in humans, mediated by changes in DNA methylation, thereby suppressing tumour progression, decreasing inflammation, and influencing oestrogen metabolism [13].

Many environmental factors have been investigated for their possible role in the development of autoimmune disease. Dietary habits have long been implicated in the development of diseases such as hypertension, heart disease, and cancer [14,15,16]. Specifically, certain beverages, such as sugar-sweetened drinks, have been found to be associated with higher rates of diseases including stroke, hypertension, and chronic kidney disease [17,18,19].

### 1.2. Diet and the Microbiome

However, despite Shoenfeld and Isenberg’s early suggestion of the role of environmental factors, such as nutrition, in the pathogenesis of autoimmune disease, it remains a relatively unexplored area, only now coming to the fore as we move towards an era of digitisation and personalised medicine.

An important factor alongside diet and nutrition is the human gut microbiome, comprising bacteria in the order 10^13^ living in symbiosis with their host [20,21]. It responds quickly and dramatically to changes in available nutrients. For example, consumption of purely animal or plant-based products for 5 days significantly alters the microbiome [22], and our habits of beverage consumption are likely to have similar effects. One of the earliest sources of nutrition for humans is breast milk, which has been shown to be critical in laying the foundations of the infant gut microbiome [23]. As we grow and our diet diversifies, beverage consumption has implications for our gut microbiota. High consumption of sugars such as fructose, found in juices and carbonated drinks, can reduce the density of beneficial gut bacteria, as demonstrated in both animal and human studies [24,25]. Chronic caffeine consumption also alters the microbiome, although it is unknown to what extent these changes are beneficial or harmful [26,27].

**Figure 1:**
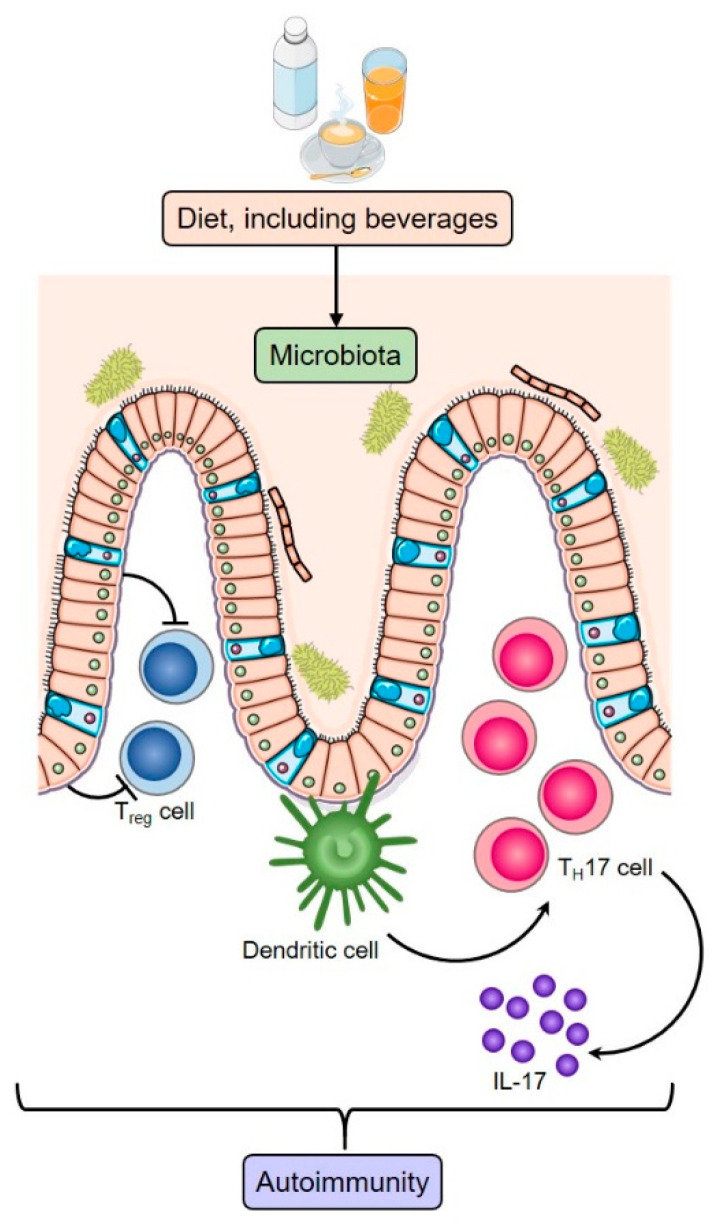
Nutritional factors found in beverages can alter the composition of the human gut microbiome, resulting in dysbyosis. Interactions between gut microbiota and the immune system are complex, involving many molecular mechanisms. An example is suppression of anti-inflammatory regulatory T (T_reg_) cells and induction of T helper 17 (T_H_17) cell differentiation, which can increase susceptibility to autoimmune diseases. (Adapted from [9])

The microbiome has been implicated in multiple autoimmune diseases, including RA (Figure 1). Dysbiosis has been observed in patients prior to the onset, or at diagnosis, of RA, with subsequent partial resolution following treatment [28,29,30]. In particular, enrichment of members of the Prevotellaceae bacterial family, particularly *Prevotella* spp., has been found to be associated with RA onset [31].

Whilst the gut microbiome provides one plausible aetiological explanation for beverage consumption in RA, the role of beverages is far more complex and deserves a more in-depth review.

## 2. Beverages in the Context of Wider Dietary Patterns

### 2.1. Western Diet

Dietary patterns, through modifications of the gut microbiome, are able to influence innate immune activity through altered production of pro-inflammatory cytokines, as we have discussed. It follows, therefore, that beverages should be enjoyed as part of a “healthy” diet. However, what form should this healthy diet take? The association between certain diets and systemic markers of inflammation has been demonstrated in both animal models and humans. For example, mice given a high-fat, high-sucrose “Western diet” develop dysregulated bile acid synthesis with increased systemic inflammation, as well as increased levels of pro-inflammatory cytokines, such as interferon (IFN)-γ and TNFα [32,33]. In addition, the Western diet classically features a high content of sugar-sweetened drinks, not only carbonated but also in sweetened coffees and cocoa-based products, which, as discussed, have been shown to have pro-inflammatory effects and contribute to poor clinical and patient-reported outcomes in inflammatory arthritis. Additions of high levels of sugar to beverages such as coffee, for example, may negate any potential beneficial effects of this drink.

### 2.2. Mediterranean Diet

The Mediterranean diet (MD) has gained popularity in recent years for its proven substantial health benefits, including in RA [34]. It was conceived prior to the 1960s, before the globalisation of dietary habits and subsequent influences on lifestyle. Rich in oleic acid, omega-3 fatty acids, unrefined carbohydrates, and phytochemicals, the MD is characterised by predominantly plant foods, such as cereals, fruits, vegetables, nuts, and seeds, with olive oil as the principal source of fat; low–moderate amounts of dairy products, fish, and poultry; a maximum of four eggs per week, and low amounts of red meat. Wine is consumed at low–moderate amounts, usually with meals, conferring the beneficial effects of this beverage, as discussed above. A hallmark of the diet is its low levels of saturated fats [35]. Adherence to the MD has been linked to significant reductions in mortality and morbidity, including in diseases such as cardiovascular disease, type 2 diabetes mellitus, obesity, degenerative diseases, as well as inflammatory diseases [36,37,38,39].

Many studies have now been conducted which demonstrate the benefits of the MD for improved disease outcomes in RA. A large population-based case-control study showed good adherence with the MD reduced the odds of developing RA by 21%, albeit in males and seropositive RA only [40]. A separate large-scale prospective study also failed to show a significant association between Mediterranean diet and risk of RA in women [41]. However, a smaller randomised control trial examining the effects of the MD in combination with a dynamic exercise programme in women demonstrated some improvement in quality of life in patients with RA with low disease activity taking conventional synthetic disease-modifying anti-rheumatic drugs (DMARDs) [42], which may suggest greater benefits of the MD in combination with other lifestyle factors. The positive effects on RA disease activity seen with the MD are likely due to high proportions of monounsaturated fatty acids (MUFA) and ω3-polyunsaturated fatty acids (PUFA), with regular intake leading to greater likelihood of disease remission [43,44]. A rich source of ω3-PUFA is fish oil, which has been shown to improve tender joint count and grip strength, with decreased use of non-steroidal anti-inflammatory drug (NSAID) use in RA patients [45,46,47]. The beneficial effects of fish oil are dose and duration dependent, as demonstrated in a small trial of patients consuming low-dose ω3-PUFA, high-dose ω3-PUFA, and olive oil supplements [48]. Improvements in clinical RA manifestations were seen across all three study groups, but reductions in swollen and tender joint count were only seen in those taking fish oil, with physician and patient pain scores improving only on consumption of high-dose ω3-PUFA.

The benefits of the MD are also attributable to the high polyphenol content, as found in fruit juices, discussed previously. Juices, as well as low-moderate amounts of wine, certainly appear to be two beverages which would complement the benefits from a rounded MD.

### 2.3. Vegetarian and Vegan Diets

Plant-based diets have also gained popularity recently and allow for consumption of most of the beverages discussed here. Increased consumption of fibre as part of this diet can improve the gut microbiome, increase bacterial diversity, and reduce inflammation and arthralgia [49]. An early study of twenty-seven patients showed significant clinical improvement after a period of following a vegetarian diet [50]. More recently, RA patients on a vegan diet have been shown to have greater ACR20 improvement and reduced immunoreactivity, as well as lower circulating levels of harmful low-density lipoprotein, and upregulation of atheroprotective natural antibodies, of importance in RA due to increased risk of cardiovascular co-morbidities in this population [51,52]. Beverages such as fruit juices and tea, enjoyed as part of such diets, are only likely to enhance these beneficial effects, alongside pharmacological therapy.

### 2.4. The Impact of Fasting on RA Disease Outcomes

Fasting, a period during which one abstains from all or some forms of food and drink, is integral to many cultures and religions, and therefore worthy of mention. Inflammation and pain have both been shown to decrease after periods of fasting, with inflammation returning on resuming normal diet, unless vegetarian, as described above [50,53,54]. Several studies have been conducted in patients observing Ramadan (a period of intermittent fasting undertaken by Muslims), with mixed outcomes in RA. Whilst one recent study showed clinical benefits in patients with RA who were observing Ramadan, another demonstrated these improvements in a non-fasting as well as fasting cohort of RA patients [55,56]. Fasting has also been explored at the molecular level, leading to decreased levels of IL-6 and disease activity [57]. IL-6 has diverse roles in autoimmune disease and alterations in its expression affects downstream disease activity [58]. IL-6 has pleiotropic biological activities, including anti-inflammatory properties under certain conditions [59]. However, in RA, its overproduction, particularly in the synovial cells and macrophages in joints, correlates with increased disease activity and joint damage. IL-6 also contributes to and facilitates maintenance of autoimmunity via B cell modulation and Th17, as well as enhancing angiogenesis via intracellular adhesion molecules [60,61,62]. Of note, in relation to diet, IL-6 is also associated with the development of metabolic syndrome and cardiovascular disease in patients with RA, as demonstrated by the administration of IL-6 inhibitor, tocilizumab, leading to a subsequent increase in insulin sensitivity [63,64]. This may provide one explanation for the association between fasting and decreased IL-6 and subsequent decreased disease activity. While this is an area that deserves further research, there is evidence to suggest that fasting can have benefits in RA at both the molecular and clinical level.

## 3. An Evolving Multidisciplinary Team: The Importance of the Dietician

Dietary factors, including the nutritional contents of beverages, are an important aspect of patient care to consider as an adjuvant to pharmacological therapy and psychosocial support. It has been recognised for decades that patients appreciate educational input from dieticians. In fact, in one early study, patients with RA rated the input of dieticians and nutritionists above that of the physician [65]. Despite the clear need for dieticians, from both the perspective of the patient and the impact of nutrition on disease outcomes, this has not translated to widescale integration of dieticians in the multidisciplinary team. A recent cross-sectional study in the UK revealed that only 17% of departments had multidisciplinary teams comprising all professional groups, although this did not look at dieticians or nutritionists specifically [66]. A separate study conducted by the Scandinavian Team Arthritis Register-European Team Initiative for Care Research (STAR-ETIC) showed consistency in composition of multidisciplinary teams across Sweden, The Netherlands, Denmark, and Norway, but with variable provision of nutritionists or dieticians [67]. Given the increasing body of data for nutritional implications on RA disease outcomes, it is necessary to increase the representation of specialist allied health professional in our holistic care of patients.

The evolving body of research of the role of individual beverages and their nutritional content in RA is discussed is discussed in depth below.

## 4. The Role of Water and Fresh Fruit Juices

Water is the most abundant of all beverages and essential to normal human homeostasis. Despite the paucity of research on water consumption in the onset and prognosis of RA, the health benefits of drinking water and adequate hydration are numerous due to the rich nutritional and mineral content (Table 1).

A greater body of research has focussed on the consumption of various fruit juices in the context of RA. Fruits, especially those rich in polyphenols, have been shown to be beneficial for health due to their antioxidant and anti-inflammatory properties [68,69]. Pomegranate juice, which has a high concentration of polyphenols, has been shown to have beneficial effects in diseases such as diabetes, atherosclerosis, and other metabolic disorders [70,71,72,73]. However, the therapeutic potential of pomegranate juice has also been demonstrated in inflammatory disorders such as inflammatory bowel disease, as well as RA [74]. A small study of fifty-five patients with RA showed ingestion of pomegranate extract for eight weeks to lead to significant reductions in disease activity score (DAS28), pain scores, and erythrocyte sedimentation rate (ESR) compared to those who did not take pomegranate extract. Reductions in health assessment questionnaire (HAQ) scores and early morning stiffness were also observed [75].

The antioxidant properties of dietary carotenoids, such as those found in orange juice, may also protect against oxidative damage in inflammatory disorders. A prospective population-based study of more than 25,000 individuals studied the effect of the consumption of carotenoids, zeaxanthin and beta-cryptoxanthin, on the development of inflammatory polyarthritis. Results showed that a modest increase in beta-cryptoxanthin intake (equal to one glass of freshly squeezed orange juice per day) was associated with a reduced risk of developing inflammatory disorders, including RA [76].

Betalain pigments within nitrate-rich red beetroot have a variety of beneficial properties in inflammatory conditions including reducing oxidative stress [77]. In the rheumatic diseases, this has recently been demonstrated in Raynaud’s phenomenon (seen in appropriately a fifth of patients with RA), with marked reductions in pan-endothelin and blood pressure, as well as pro-inflammatory cytokines, on administration of beetroot juice [78,79]. A small cohort study of patients with RA also demonstrated significant improvements in endothelial function following two weeks of daily beetroot juice consumption [80]. Betalain compounds has also proved beneficial in improving patient-reported outcomes in individuals with knee discomfort [81]. Patients taking 50 mg oral betalain-rich red beet concentrate reported a 27% better McGill Pain Questionnaire score than those taking placebo, after ten days. Knee function, as measured by the Western Ontario and McMaster Universities Arthritis Index (WOMAC) score, was 26% better in patients taking betalain compared to placebo. Finally, energy levels, as reported using a visual analogue score, were significantly better at both five and ten days in the patients taking betalain supplementation. These results suggest potential benefits for patients with joint pain in RA.

Cranberry juice is another beverage known to have antioxidant effects, with studies demonstrating clinical benefits in conditions including hypertension and type 2 diabetes mellitus [82,83]. In RA, a small prospective study of 41 women demonstrated a decrease in DAS28 and anti-CCP titres after 90 days of consumption of low-calorie cranberry juice. While these findings should be replicated in a larger cohort, they certainly suggest cranberry juice may have yielded therapeutic effects alongside conventional medications in patients with RA [84].

## 5. Tea and Coffee

Tea and coffee are two beverages which are enjoyed in abundance around the world. With more than one hundred varieties of coffee, and over 20,000 varieties of tea, these drinks are integral to many cultures, each with their own unique nutritional profile.

These beverages have multiple effects on human health through a variety of mechanisms. As mentioned previously, dietary phenols in both tea and coffee are able to alter DNA methylation, with downstream effects on factors including inflammation and oestrogen metabolism [13,85]. However, the anti-inflammatory properties of these beverages span far beyond the epigenome.

**Figure 2:**
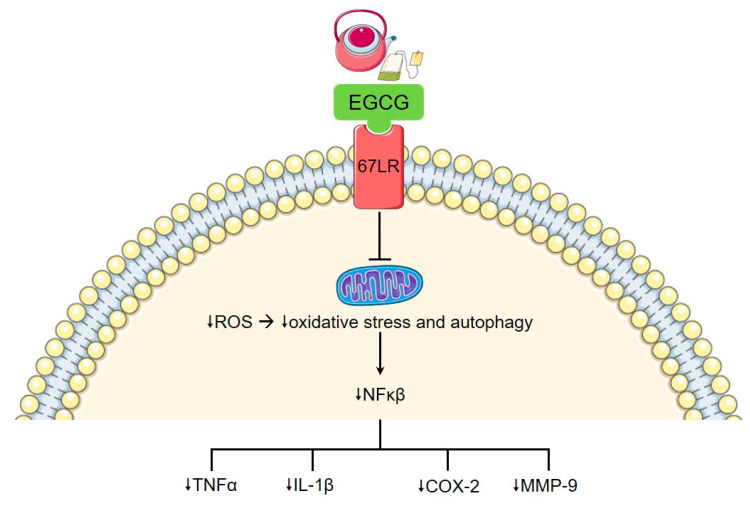
Effect of epigallocatechin-3-gallate (EGCG) on inflammation. EGCG modulates intracellular signalling pathways via the 67 kDa laminin receptor (67LR). This reduces mitochondrial production of reactive oxygen species, leading to reduction in pro-inflammatory cytokine production, including NFκβ. This has multiple downstream effects, including downregulation of TNFα, IL-1β, cyclo-oxygenase-2 (COX-2), and matrix metalloproteases (MMP), such as MMP-9.

Epigallocatechin-3-gallate (EGCG), the main phytochemical and flavonoid-containing catechin present in green tea (*Camellia sinensis*), has gained significant attention in recent years in areas as wide ranging as anti-angiogenesis in the setting of malignancy to cardiovascular disease [86,87]. Protective effects in the setting of autoimmune disease have also been studied. EGCG inhibits CD4+ T cell expansion in response to polyclonal or antigen-specific stimulation, as well as impeding Th1 and Th17 differentiation through downregulation of their transcription factors (STAT1 and T-bet for Th1, and STAT3 and RORγt for Th17), corresponding with reduced clinical symptoms in mouse models of multiple sclerosis [88] (Figure 2). The balance of Th17 and Treg is crucial in the pathogenesis of RA, which may partly explain the benefits of EGCG consumption in this disease. Green tea extract reduces chemokine production in RA synovial fibroblasts leading to decreased inflammation, as well as modestly ameliorating adjuvant-induced arthritis in rat models [89]. In addition, the catechin epigallocatechin gallate (EGC), works synergistically with EGCG to inhibit IL-6, IL-8, and MMP-2 production and selectively inhibits COX-2 expression [90]. However, not all catechins in green tea appear to be equal, as demonstrated by epicatechin (EC), which, in this same study, was shown to hinder the marked downstream inflammatory signalling inhibition exerted by EGCG.

There is a relative paucity of data on the effect of other forms of caffeinated tea on RA disease trajectory. However, a large longitudinal prospective cohort study conducted in women between 1993 and 1998 did demonstrate a small positive association between daily caffeinated, non-herbal tea consumption and incident RA [91].

The therapeutic role of coffee in RA remains less clear. Data has long been conflicted when investigating a correlation between coffee consumption and mortality [9]. Early studies suggested an association between coffee consumption and cardiovascular-related death [92,93]. However, most studies were based on observational data, and one needs to be aware that association does not equal causation. More recent data suggests an inverse correlation between coffee consumption and mortality, with moderate coffee consumption (e.g., 2–4 cups per day) associated with reduced all-cause and cause-specific mortality, compared to no coffee consumption [94].

When considering the effect of coffee in RA onset and progression, caffeine is the main ingredient of interest, which has been demonstrated to have immune-modulatory and anti-inflammatory properties. Caffeine alters intracellular calcium-signalling patterns in lymphocytes, important for their activation and effector function. It is able to suppress tumour necrosis factor (TNF) in mouse and human studies, with decreased lymphocyte proliferation and antibody production, in part due to caffeine’s inhibition of cyclic adenosine monophosphate (cAMP) phosphodiesterase [95,96,97].

However, there is a marked difference when looking at coffee in the context of rheumatic diseases. While this beverage exerts a protective role in the development of certain autoimmune diseases including multiple sclerosis and ulcerative colitis, consumption appears to increase the risk of developing seropositive RA, although this is not seen in seronegative RA or in consumption of decaffeinated coffee [98,99]. Consistent with this, one cross-sectional study found that the number of cups of coffee drunk daily was directly proportional to the prevalence of RF positivity, although this has yet to be repeated in a larger cohort, and may have been confounded by factors such as active or passive smoking in this population [100]. Of note, as a methylxanthine and adenosine receptor antagonist, caffeine has been shown in several studies to decrease the efficacy of methotrexate, the most commonly prescribed disease-modifying anti-rheumatic drugs (DMARD) in the treatment of RA [101,102]. One small study of 39 patients specifically found >180 mg/day caffeine interferes with the efficacy of methotrexate in patients with RA, when compared with patients consuming <120 mg/day [102]. More recent work has implicated the role of certain mutations of the ADOARA2A gene in patients at risk of decreased efficacy of methotrexate when consuming large quantities of caffeine, while other genotypes configure significant protection against this effect [103].

It is of course important to note the other effects of high caffeine intake, such as palpitations and poor sleep, in addition to sugars and high-fat dairy products present in some caffeinated drinks; it is thus best consumed in moderation.

## 6. Cocoa-Based Beverages and Milk

Cocoa has traditionally been considered therapeutic, with direct and indirect antioxidative properties having positive benefits for hypertension, insulin resistance, and cardioprotection [104,105,106]. While green tea is abundant in the polyphenol catechin, cocoa is a rich source of the polyphenol, flavanol, giving rise to its antioxidative and anti-inflammatory properties. In cellular and animal models of inflammatory arthritis, cocoa specifically reduces expression of cytokines such as interleukin (IL)-2 and TNFα, as well as reducing production of reactive oxygen species and nitric oxide from macrophages [107,108]. However, large-scale human studies are lacking in this area, rendering the therapeutic potential of cocoa in RA under continued debate.

A common accompaniment to tea, coffee, and cocoa drinks is milk. An early study, investigating ingestion of cow’s milk in rabbits, noted the development of rheumatoid-like lesions in 36% of the cohort after 12 weeks. Specifically, an increased number of nucleated cells and raised percentage of T lymphocytes were seen in the synovial fluid, correlating with severity of histological lesions [109]. However, a more recent study on IL-1Ra-deficient mice and collagen-induced arthritis demonstrated bovine milk derived extracellular vesicles (BMEVs) from semi-skimmed milk delayed onset of arthritis and reduced cartilage pathology and bone marrow inflammation. Serum levels of MCP-1 and IL-6 as well as Th1 and Th17 were also diminished [110]. Similar results have been seen on administration of camel’s milk to rat models with adjuvant-induced arthritis, with downregulation of TNFα and upregulation of anti-inflammatory IL-10 [111]. IL-10 is produced by almost all innate and adaptive immune cells, with highly regulated secretion and action. Whilst it has been considered pathogenic in certain autoimmune conditions, such as systemic lupus erythematosus, due to promotion of humoral immune responses via B cell modulation, it is considered anti-inflammatory in RA pathogenesis [112]. Preclinical studies in animal models, including collagen-induced arthritis, show IL-10 to effectively inhibit inflammation. Exogenous addition of IL-10 in vitro also has beneficial effects of immunopathology in RA. Preliminary studies of human recombinant IL-10 in patients with RA have a good safety profile, although harnessing this in therapeutics has proven difficult. Upregulation of IL-10 via other exogenous sources, including beverages such as milk, may therefore prove a useful alternative [113].

Human studies investigating the overall effects of milk in RA are limited in number and have produced mixed results. Data from the Swedish Mammography Cohort showed no association between the total consumption of milk and dairy products over 12 years, and the risk of developing RA [114]. However, a large case-control study demonstrated slightly increased likelihood of developing RA when drinking full-fat milk (odds ratio 1.01) [115]. Of note, when considering milk allergy, concentrations of milk-specific IgE and IgG have been shown to be enhanced in collagen-induced arthritis rat models, suggesting a close correlation with the pathogenesis of RA [116].

Data is therefore limited and contradictory, but milk does provide vital nutrients such as vitamin D, calcium, and protein, and choice and amount of consumption remains down to the individual.

## 7. Sugar-Sweetened Soft Drinks

Almost all of the beverages discussed thus far contain high levels of sugars, both natural and added. Negative impacts of prolonged high sugar consumption on health are well documented [117]. Sugar-sweetened drinks in particular are associated with increased prevalence of obesity and obesity-related diseases, such as type 2 diabetes mellitus and cardiovascular disease [118,119,120]. High glucose intake exacerbates autoimmunity in mouse models of colitis and experimental autoimmune encephalomyelitis, through the activation of transforming growth factor (TGF)-β and promotion of Th17 cell differentiation [121]. Increased intake of glucose and fructose appears to have similar effects in humans, with reports suggesting overactivation of the mTOR pathway [122].

Few studies have been performed in the setting of RA, but are consistent with these findings, affecting both onset of disease, as well as disease progression. A large-scale epidemiological study based on the Nurses’ Health Study (NHS) initiated in 1976, with over 121,000 female registered nurses, demonstrated regular consumption of sugar-sweetened soda, but not diet soda, is associated with increased risk of seropositive RA in women, independent of other dietary and lifestyle factors [123]. Self-reported outcomes by patients have also been found to worsen with long-term consumption of sugar-sweetened soda [124].

This association may in part be due to excess free fructose (EFF), which when unabsorbed, contributes to formation of advanced glycation end-products (enFruAGEs), a source of pro-inflammatory advanced glycosylation (AGEs) end-products, as found in the caramel component of soda. These unabsorbed components accumulate in other tissues, such as synovial tissue, potentially contributing to autoimmune arthritis. The effect appears to be particularly marked in young adults. One cross-sectional study in the United States showed 20–30-year olds consuming any combination of high EFF beverages ≥5 times per week (but not diet soda) were three times as likely to have inflammatory arthritis, independent of factors such as physical activity, other dietary components, blood glucose, and smoking [125]. It is therefore likely that, at least in young adults, regular excess consumption of sugar-sweetened beverages, especially those containing fructose, poses an increased risk of RA. Taken with the excess risk of cardiovascular co-morbidities in these patients, decreased consumption of such beverages may infer positive health benefits in this population.

## 8. Alcohol Intake

While no official guidance exists on alcohol consumption in RA, several studies has been conducted in this area. UK guidelines recommend a maximum of 14 units per week of alcohol for men and women, in which a unit comprises 10 mL alcohol. A meta-analysis of a total of 195,029 participants, including 1878 RA cases, showed low–moderate alcohol consumption to be inversely associated with development of RA in a dose-dependent, time-dependent, and sex-dependent manner [126]. Women with low–moderate alcohol consumption had a 9% reduction in RA risk, and, regardless of gender, consistent low–moderate alcohol intake for at least 10 years led to a 17% reduction in risk. Of note, a study of 596 patients, suggested moderate consumption of alcohol leads to increased radiographic progression in women, but not men, with early RA [127].

**Figure 3:**
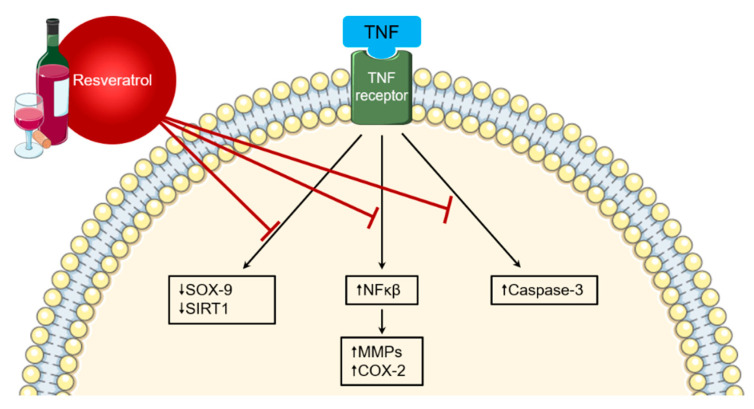
Anti-inflammatory properties of resveratrol. Resveratrol has been shown to inhibit several pro-inflammatory pathways, reducing expression of factors such as NFκβ and caspase-3. It also leads to upregulation of anti-inflammatory molecules including SOX-9 and SIRT1.

A particular chemical of interest for its antioxidant and anti-inflammatory properties is resveratrol, found in high concentrations in red wine [9]. Resveratrol exerts its immunomodulatory effects in numerous ways, including the suppression of TNF-induced nuclear factor (NF)-κβ, prostaglandins, and COX-2 [128,129] (Figure 3). In rat models of antigen-induced arthritis, resveratrol significantly reduces pathological and clinical hallmarks of RA, such as knee swelling and histological scoring of synovial tissue. This is associated with significantly decreased proliferating cell nuclear antigen (PCNA), CD68, CD3, monocyte chemoattractant protein-1 staining, ROS, and DNA damage [130,131]. Clinical studies in humans are few in number but appear to support the consumption of resveratrol as an adjuvant to conventional pharmacological therapy in RA. A randomised controlled trial of 100 patients with RA demonstrated that those receiving a daily resveratrol capsule of 1 g with conventional treatment for 3 months had significantly decreased clinical markers, DAS28 scores, and biochemical markers (e.g., C-reactive protein and ESR), compared to those who did not take resveratrol with regular therapy [132].

Despite these positive findings, it is important to take this in the context of known significant harmful effects of alcohol, and hepatic toxicity risks with some RA medications, such as methotrexate and sulfasalazine. Therefore, there is no evidence to suggest patients increase their resveratrol intake through increased red wine consumption (for example).

## 9. The Role of Beverages during Treatment of RA

Beverages should be considered as one element of the wider diet, as well as the inherent dietary disturbances and gastrointestinal side-effects that can arise from various aspects of RA and its management. Many of our most effective and commonly used DMARDs, such as methotrexate, can have adverse gastrointestinal side effects including diarrhoea and nausea [133]. This can have wider implications for malnutrition, overall well-being, and disease activity outcomes. The widespread and often prolonged use of glucocorticoids corticosteroids brings with it multifaceted effects on the gastrointestinal system, including increased risk of gastrointestinal bleeding and perforation, nausea and vomiting, and effects on metabolism leading to weight gain, hyperglycaemia, and type 2 diabetes mellitus [134,135]. In fact, patients on long-term corticosteroids are four times as likely to develop type 2 diabetes mellitus, compared to controls. This highlights the need to consider diet, especially aspects such as sugar intake, in the holistic management of RA patients. As detailed here, many beverages have a high sugar content, with proven harmful effects for both disease onset and progression.

## 10. Conclusions

In general, beverages are integral to our lives, whether for rehydration, socialising, or as one element of our rich and varied diets. However, with a surprisingly high and diverse nutritional contents, beverages can contribute many benefits for patients with RA, although in some cases may do more harm than good. In fact, as with all food groups, some yield more positive effects than others, and it is particularly important to be aware of the high sugar content of some beverages, especially given the potential for poor cardiovascular outcomes and features of metabolic syndrome in RA patients. Nonetheless, some of our favourite beverages, such as tea, fresh fruit juice, and even moderate amounts of red wine, can confer benefits at the molecular, epigenetic, and clinical level when taken in conjunction with traditional therapies. The growing evidence base for correct nutrition in RA and global health benefits of a healthy diet highlights the need for improved access to dietary counselling for patients, whether this be with a dietician or the physician. In this regard, it is promising that large organisations, including the European League Against Rheumatism (EULAR), have recognised the importance of diet and nutrition in RA, leading to the development of new educational initiatives such as a recent teaching module for online courses dedicated to nutrition and rheumatic diseases [136]. Increased patient and clinician awareness of the role of beverages in RA has the potential to improve the dietary and lifestyle guidance we are able to provide, with positive impacts for the holistic management of patients.

## Figures and Tables

**Table 1:** Dietary reference values for carbohydrates, dietary fibre, total fat, fatty acids, protein and water.

Nutrient	Age Range (Years)/Physiological State					
	≥18	Pregnancy			Lactation	
		1st Trimester <12 Weeks	2nd Trimester 13 < 28 Weeks	3rd Trimester ≥ 28 Weeks	0–6 Months Post-Partum	>6 Months Post-Partum
Total carbohydrates ^a^ (E%)	45–60					
Dietary fibre ^b^ (g/day)	25					
Total fat ^a^ (E%)	20–35	20–35			20–35	
SFA	ALAP	ALAP			ALAP	
LA ^b^ (E%)	4	4			4	
ALA ^b^ (E%)	0.5	0.5			0.5	
EPA + DHA ^b^ (mg/day)	250	250			250	
DHA ^b^ (mg/day)		+100–200 ^c^			+100–200 ^c^	
TFA	ALAP	ALAP			ALAP	
Protein						
AR ^d^	0.66	+0.52 ^e^ g/day	+7.2 ^e^ g/day	+23 ^e^ g/day	+10 ^e^ g/day	+15 ^e^ g/day
PRI ^d^ (g/kg bw/day)	0.83	+1 ^f^ g/day	+9 ^f^ g/day	+28 ^f^ g/day	+19 ^f^ g/day	+23 ^f^ g/day
Water ^b,g^ (L/day)						
Males	2.5					
Females	2.0		2.3		2.7	

ALA, a-linolenic acid; ALAP, as low as possible; AR, average requirement; DHA, docosahexanoic acid; EPA, eicosapentaenoic acid; E%, percentage of energy intake; L, litre; LA, linoleic acid; PRI, population reference intake; SFA, saturated fatty acids; TFA, trans-fatty acids. ^a^ RI, reference intake range, ^b^ AI, adequate intake, ^c^ in addition to combined intakes of EPA and DHA of 250 mg/day, ^d^ to be multiplied by reference body weights to calculate values in g/day, ^e^ in addition to AR for protein of non-pregnant, non-lactating women, ^f^ in addition to PRI for protein of non-pregnant, non-lactating women, ^g^ includes water from beverages of all kind, including drinking and mineral water, and from food moisture. (European Food Safety Authority, 2017. Based on Cutolo, M. and Nikiphorou, E. 2019 ‘EULAR Online Course on Rheumatic Diseases: Nutrition in Rheumatic Diseases’, Module 42d.).
